# Comparison of the chemical and micromechanical properties of *Larix spp*. after eco-friendly heat treatments measured by *in situ* nanoindentation

**DOI:** 10.1038/s41598-020-61314-6

**Published:** 2020-03-09

**Authors:** Dong Xing, Jian Li, Siqun Wang

**Affiliations:** 1 College of Materials Science and Art Design, Inner Mongolia Agriculture University, Hohhot, 010018 China; 2 College of Materials Science and Engineering, Northeast Forestry University, Harbin, 150040 China; 3 Center for Renewable Carbon, University of Tennessee, Knoxville, TN 37996 USA

**Keywords:** Cell wall, Nanometrology

## Abstract

Heat treatment is a green, environmentally friendly and mild pyrolysis process that improves the dimensional stability and durability of wood. In this study, *Larix spp*. Samples were heated at 180 °C and 210 °C for 6 h with nitrogen, air or oil as heat-conducting media. The influence of high-temperature heat treatment on the microstructure, chemical components, and micromechanical properties was investigated. The mass loss rate increased with increasing temperature, and the degradation of wood components resulted in cracks in the cell walls. Samples treated with air showed more cracks in cell walls than were observed in the cells walls of wood treated with the other heat-conducting media. The hardness of the cell walls increased after all heat treatments. In addition, the results showed that heat treatment reduced creep behavior compared to that of untreated wood.

## Introduction

*Larix spp*., an appealing source of wood products in China, are massively planted due to their excellent adaptability, rapid growth and long growth cycle. However, the poor dimensional stability and durability of these species severely limits their scope of applications^1^. Recently, several industrial-scale and environmentally friendly heat treatment processes were applied to solve these problems without the addition of chemical agents, including Retification^®^, Thermowood^®^, Plato Wood^®^, OHT^®^ and so on^2^. The equilibrium moisture content (EMC) and hygroscopicity of wood decreased markedly after heat treatment, which could prevent several appearance defects, such as blue staining and white rot. Moreover, after appropriate heat treatment, wood durability can be enhanced significantly, which extends the life cycle of the wood products accordingly^3^.

Heat treatment, a green process, alters the chemical composition of wood through pyrolysis of the hemicellulose and part of the amorphous cellulose, evaporation of extractives and condensation of byproducts^4,5^. This procedure caused a higher degree of cellulose crystallinity, lower hydrophilicity, improved color, and better dimensional stability and durability of wood^6,7^. The mechanical properties of heat-treated wood, including compression strength, modulus of elasticity, modulus of rupture, brittleness, impact bending and so on, have been extensively reported on^8^. Previous studies have shown that the elemental composition (C/O ratio or C content) is a useful marker for predicting the level or extent of heat treatment^9,10^. These markers can also be effectively used for the prediction of decay durability and mechanical properties of heat-treated wood. Furthermore, the mass loss rate is also a notable feature in heat treatment and is a parameter used for quality control^11,12^. It has been massively proven that chemical transformations of compounds and extractives in wood cell walls is caused by heat treatment^13,14^. These transformations are mainly dependent on the wood species, treatment duration, heat temperature and so on^15–17^.

If wood heat treatment involves air on an industrial scale, a lower quality and higher risk of burning are inevitable. Oil heat treatment is a new approach used in commercial wood modification processes and requires large equipment investments, complex technology and high consumption of vegetable oil^18^. Nitrogen is also used as a heating medium for heat treatment; the equipment required for this needs good, gas-tight seals, and the products represent relatively higher brittleness than that of the untreated wood^19^. In general, the selection of a heating medium is the main process condition of wood heat treatment technology, and it has an important influence on wood performance. However, there are few publications regarding the influence of different heat media or atmospheres on the properties of heat-treated wood.

At the macrostructure and ultrastructure scales, the mechanical properties of wood are significantly affected by heat treatment. The main objective of this study was to investigate the effect of different heating media, nitrogen, oil and air, on the chemical composition, cell wall structure and micromechanical properties of thermally modified wood.

## Experimental

### Materials

*Larix gmelinii (Rupr.)* is a plantation forest species mainly planted in northern China. A 12-year-old *Larix* was selected from Harbin, Heilongjiang Province, China. The samples were prepared from the same log with dimensions of 10 mm × 9 mm × 9 mm (longitudinal, radial and tangential dimensions). The tips of the samples were cut into a pyramidal shape and were all from the same latewood growth ring^12^. First, the samples were oven dried and then placed in a plastic bag for SEM observation and nanoindentation testing.

### Heat treatment

There were three heat treatment processes used in the experiments with three different heating mediums: nitrogen, oil and air. Wood specimens were heat-treated under nitrogen or air (20 ml/min) and were denoted as N1, N2, A1 and A2, where N represented nitrogen and A represented air. The other samples were soaked in canola oil for heat treatment and then oven dried to remove the spare oil in the wood samples. These samples were denoted as O1 and O2, with O representing oil. All samples were removed after being cooled to room temperature. The detailed parameters of heat treatment are listed in Table 1.

**Table 1:** Detailed parameters of heat treatment under different atmospheres.

Specimen No.	Holding Temp (°C)	Heat Atmosphere	Heating rate (°C/h)	Duration (h)
Untreated	—	—	—	—
N1	180	Nitrogen	15	6
N2	210	Nitrogen	15	6
A1	180	Air	15	6
A2	210	Air	15	6
O1	180	Oil	15	6
O2	210	Oil	15	6

### Mass loss

The samples were weighed before and after heat treatment on an AX205 analytical semi microbalance (Hamilton Inc., Cinnaminson, USA) scale with 0.001 mg accuracy, and the mass loss was recorded accordingly:

$$ML( \% )=100\times ({m}_{0}-{m}_{1})/{m}_{0}$$

where *m*_0_ is the mass of the specimen after traditional oven drying and *m*_1_ is the mass of the specimen after heat treatment and then oven drying.

### Characterization of wood

Four samples with dimensions of 7 mm × 7 mm × 2 mm were coated with a gold layer for 130 s, and the applied current was 23 mA. Samples were then mounted on aluminum stubs with a conductive paste. The surface morphology of the wood cell wall was measured by scanning electron microscopy (FEI, Quanta 200, Hillsboro, Oregon, USA). The chemical changes of wood specimens were measured by infrared analytical FTIR (Fourier Transform Infrared Spectrometer, Nicolet, Magna-IR 560 E.S. P, USA) before and after heat treatment.

### Nanoindentation test

Nanoindentation was used to measure all wood samples by using a diamond Berkovich tip. Samples were well prepared and tested following the procedure described by Xing *et al*. (2016).

## Results and Discussion

### Mass loss rate

**Figure 1:**
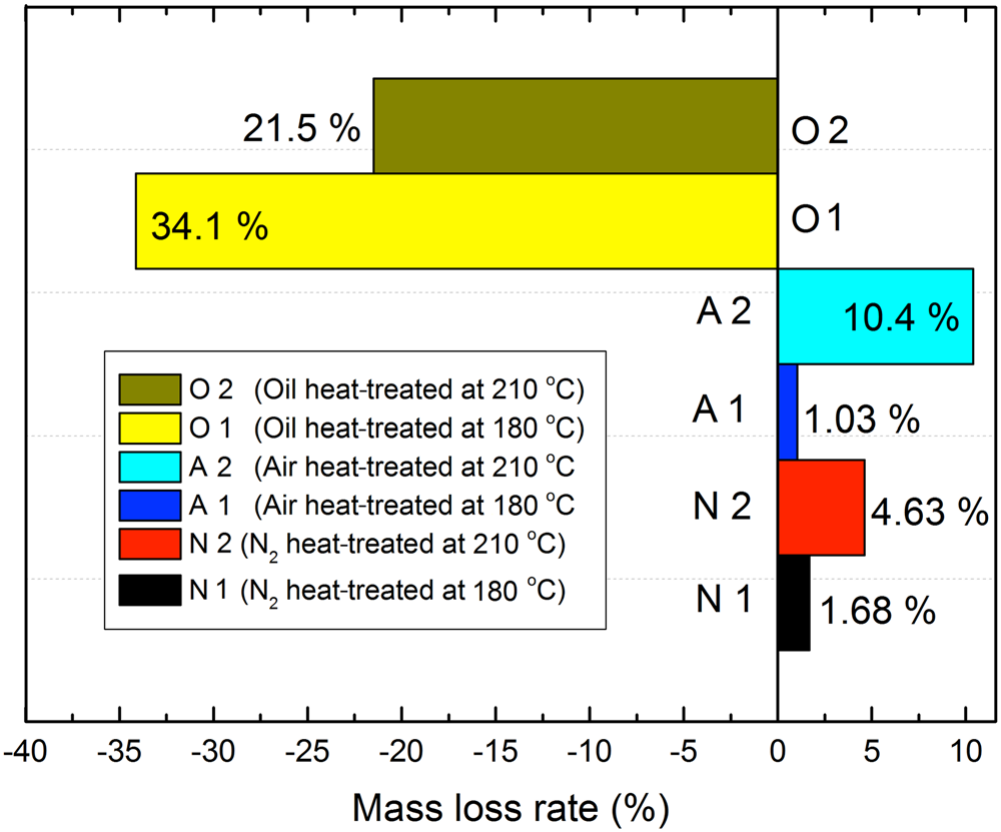
The mass loss rate of different heat-treated processes.

The degradation of wood components and the evaporation of extractives was observed since the mass of wood decreased gradually^20^. The mass loss rate showed the different amounts of hemicellulose degradation, oil uptake and the extent of heat treatment^21^. Figure 1 shows the mass loss rates of the samples heat-treated with different heating mediums. Slight mass loss rates of 1.68% and 4.63% were observed for N1 and N2, respectively, corresponding to vaporization of volatile extractives, degradation of hemicellulose and some amorphous cellulose and desorption of bound water in the wood samples^19,22,23^. The higher mass loss rate of A2 (10.4%) may have been due to the severe pyrolysis and oxidation of the cell wall components. For O1 and O2, the mass increased by 34.1% and 21.5%, respectively. This was likely caused by the oil penetrating into the cell walls, lumens and middle lamellas during oil heat treatment.

### FTIR

**Figure 2:**
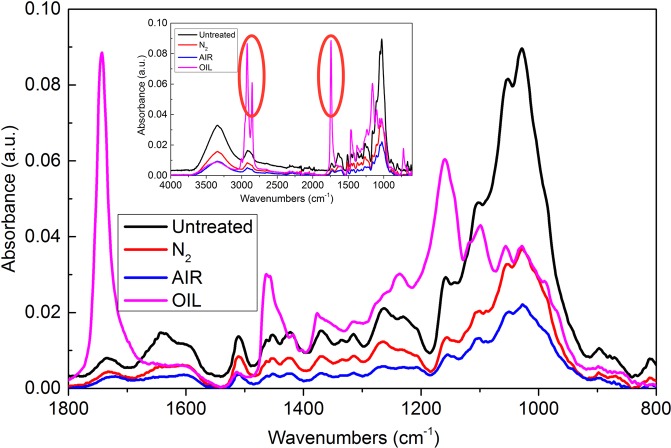
FTIR spectra of wood samples subjected to three different heat treatment processes and that of an untreated sample.

**Figure 3:**
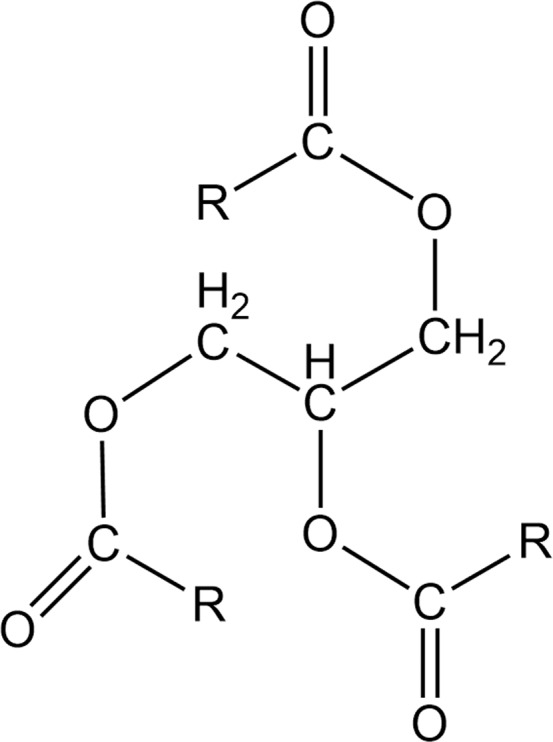
The molecular formula of triglyceride fatty acids.

**Figure 4:**
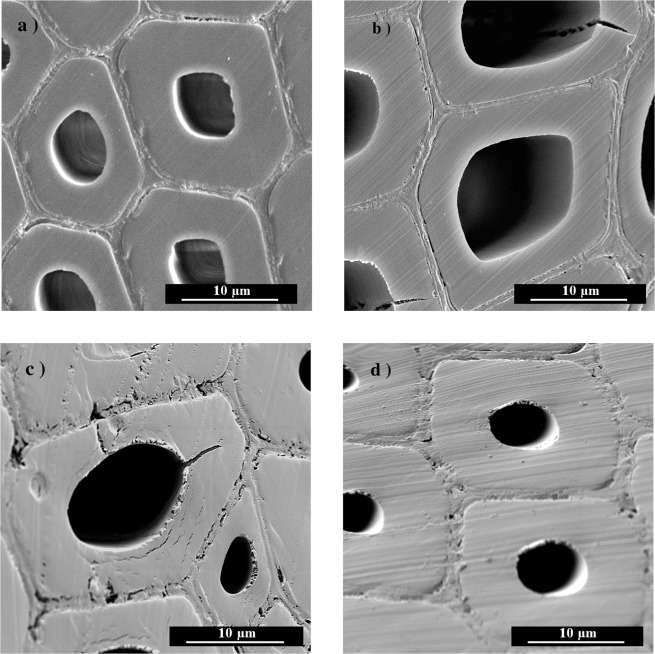
Microstructure observation of wood by SEM: (**a**) an untreated sample; (**b**) a heat-treated sample under a nitrogen atmosphere; (**c**) a heat-treated sample under an air atmosphere; and (**d**) a heat-treated sample under an oil atmosphere.

The FTIR spectra of wood specimens before and after heat treatment at 210 °C are presented in Fig. 2. Table 2 shows the characteristic bands and vibration types of samples in the region of 800–3500 cm^−1^. The FTIR spectra of the oil heat-treated sample contained several peaks, 2927 cm^−1^, 2854 cm^−1^, 1740 cm^−1^, 1267 cm^−1^ and 1163 cm^−1^, that did not appear in the spectra of other samples and are marked by red circles. These are the typical absorbance bands of triglyceride fatty acids, one of the main components of canola oil used in the oil heat treatment process shown in Fig. 3. The heat-treated wood under the N_2_, oil and air atmospheres all exhibited a broad band and low absorbance peak at 3336 cm^−1^ in their FTIR spectra, as shown in Fig. 3. This corresponded to the -OH stretching vibration, mainly belonging to polysaccharides. This was most likely caused by primary -OH oxidation, pyrolysis of acetyl groups of hemicellulose and modification of amorphous cellulose by dehydration^24,25^. Because of the breakdown of acetyl groups, hemicellulose was first degraded, as demonstrated by the decrease in the 1740 cm^−1^ peak. This caused cracks in the wood cell wall, which were also observed in the SEM images (Fig. 4). The peaks at 1604 cm^−1^ and 1509 cm^−1^ in the spectra of all wood samples (N_2_, oil and air) corresponded to the carbon skeleton vibration of lignin benzene rings^26^, and the broadened peaks indicated that the aromatic rings of lignin had relatively high structural diversity. Therefore, a greater range of frequencies was absorbed. The carbonyl band at 1267 cm^−1^ of the N_2_ and oil heat-treated samples showed relatively low intensities, which was likely due to the cleavage of acetyl groups in holocellulose or lignin. The peak at 896 cm^−1^ of the N_2_ heat-treated sample began to decrease, corresponding to pyranose stretching of holocellulose, which was caused by hemicellulose degradation^27,28^. All results demonstrated that the structure of the wood components, especially hemicellulose, had been modified after N_2_, oil and air heat treatment^29^. With an increasing presence of oxygen, the peaks further decreased in FTIR spectrum of the air heat-treated sample, which confirmed the greater extent of pyrolysis, oxidation and structural deterioration of holocellulose and lignin^30^. Wood chemical composition changes include deacetylation and depolymerization, in which the released acetic acid catalyzes complex pyrolysis reactions and thus reduces the accessible hydroxide radical groups^31–33^. The increased mobility and reactivity of lignin resulted in recondensation and cross-linking reactions forming a new lignocellulose structure^17,34^.

**Table 2:** FTIR absorbances of heat-treated and untreated *Larix spp*.^23,24^.

Wavenumber (cm^−1^)	Functionality
3336	O–H of alcohols, phenols and acids
2900	CH_2_, CH-, CH_3_
1738	C=O
1604	Aromatic ring (syringyl lignin)
1509	Aromatic ring (guaiacyl lignin)
1425	C–H and aromatic ring skeletal vibrations
1373	C–H (cellulose and hemicellulose)
1317	O–H (cellulose and hemicellulose)
1267	CO–OR (cellulose); aromatic ring ether (lignin)
1155	Carbohydrate C–O–C
1049	C–O, C–H (primary alcohol, guaiacyl)
1018	C–O–C
898	out-of-plane deformation of the pyranose ring
806	C–H

### SEM

SEM observations showed the anatomical structures of the untreated N2, A2 and O2 samples, and the SEM micrographs are shown in Fig. 4. The cross-section of the untreated sample represents the uncompromised cell wall and middle lamella. For N2, there were several small cracks of the wood cell wall in Fig. 4b, and the integrity of the cell wall was still relatively high, with only a 4.63% mass loss rate. The cell wall of A2, the air heat-treated wood sample, was strongly impacted since many cracks in the S1 and S2 layers appeared (Fig. 4c). Moreover, the compound middle lamella of A2 also obviously deteriorated, which made the sample more brittle than the untreated wood. Oxidation reactions strongly damage the structure of wood cell walls, which leads to lower mechanical properties of air-treated wood. This is part of the reason for applying heat treatment under oxygen-free atmospheres^17^. Both the mass loss and FTIR analysis proved that the oil penetrated into the wood cell wall of the O2 sample. After oil uptake and heat treatment, the image of O2 shows the uncompromised structure of the wood cell walls (Fig. 4d).

### Nanoindentation

**Figure 5:**
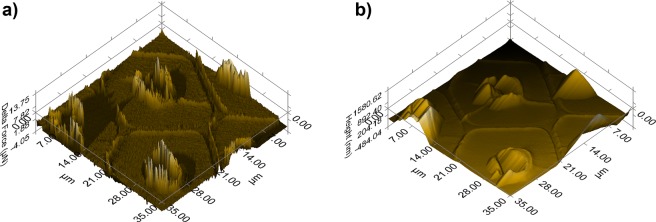
Scanning probe microscopy images: gradient 3D images and topographic 3D images.

**Figure 6:**
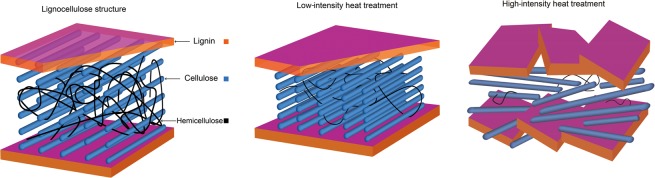
Simulated structure of wood cell walls before and after heat treatment.

Nanoindentation analysis acquires the height and force data from an analytical tip at the same time. The gathered data were used to determine the surface morphology of the wood samples by SPM (scanning probe microscopy), which is shown in Fig. 5. The gradient 3D scanning images were recorded by SPM to mark the space for indentation, which was measured in the same location and is called *in situ* nanoindentation. The gradient 3D images and topographic 3D images were also used to determine the effective data after indentation. According to previous research^8,35,36^, the changes in the cell wall structure are schematically depicted in Fig. 6. Low-intensity heat treatment was treatment that caused a tightened cellulose-lignin structure, and high-intensity heat treatment was treatment that caused cracks in the wood cell wall. Heat treatment caused hemicellulose depolymerization and densification of cellulose, which may have improved the internal bonding of the lignocellulose structure. For the high-intensity heat treatment, the breakage of hemicellulose chains, cellulose chains and modified lignin ultimately weakened the integrity of the cell wall structure and reduced the wood mechanical properties at the macroscopic level^37^, as shown in Fig. 6.

**Figure 7:**
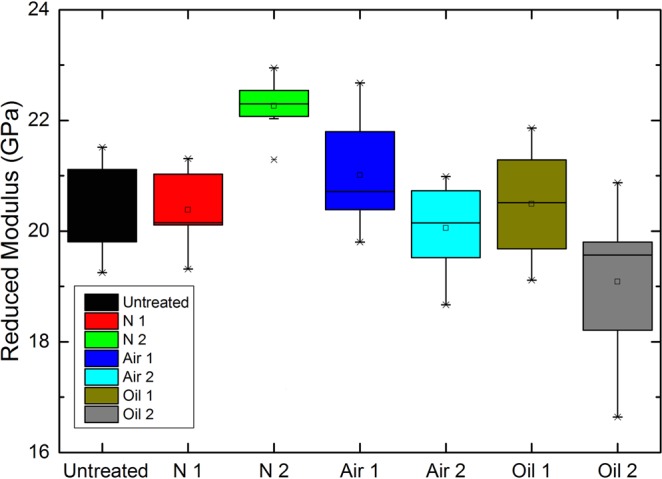
The reduced elastic modulus of the wood cell walls after heat treatment under different atmospheres.

The elastic modulus of wood varies for several reasons, such as the cellulose microfibril angle, the moisture content and the main components. Figure 7 shows the reduced elastic modulus of the heat-treated wood cell walls. The reduced elastic modulus of the S2 cell walls of N1 basically remained the same as that of the untreated cell walls. The modulus of N2 showed an obvious increase from 20.5 GPa to 22.4 GPa. This was due to the autocondensation and cross-linking reactions of the aromatic ring units of lignin during the natural cooling step^38,39^. Heat treatment has a positive influence on the micromechanical properties of wood cell walls. When oxygen was involved in the heat treatment, the elastic modulus substantially decreased from 21.0 GPa to 20.1 GPa after air heat treatment at 210 °C (A2). The greater number of oxidation reactions resulted in severe deterioration of the wood cell walls, which was also observed in the SEM micrographs. The O2 sample (oil heat-treated wood sample heated at 210 °C) showed the largest decrease in elastic modulus from 20.4 GPa to 19.1 GPa. Generally, after oil and air heat treatment, the elastic modulus of the wood cell walls decreased with increasing treatment temperature, which was due to the severe degradation of hemicellulose and damage of cellulose. For N2, the elastic modulus of the wood sample increased, which was most likely caused by the low-intensity heat treatment, which was determined by the tightened cellulose-lignin structure and the decreased moisture content after heat treatment, as shown in Fig. 6.

**Figure 8:**
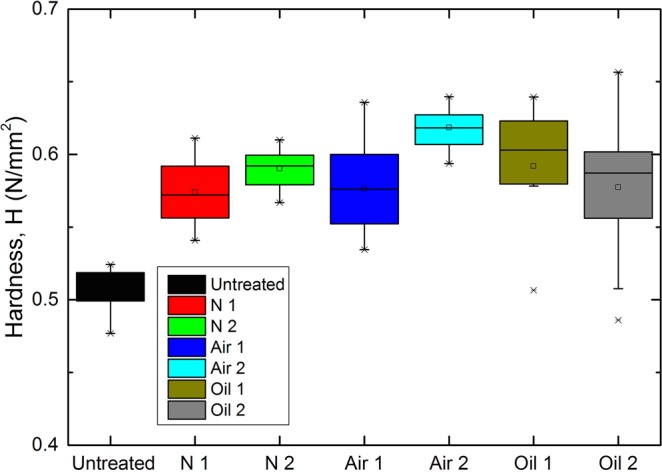
The hardness of wood cell walls after heat treatment under different atmospheres.

Figure 8 shows the hardness of call walls in the S2 layer if the samples after heat treatment. In general, the hardness of wood increased obviously after different heat treatments. The hardness of N1 and N2 increased from 0.50 N/mm^2^ to 0.57 N/mm^2^ and 0.59 N/mm^2^, respectively, which was consistent with previous research and proved that heat treatment under N_2_ conditions is a good alternative advanced manufacturing process for flooring and structural products^40,41^. The hardness of A1 and A2 increased from 0.50 N/mm^2^ to 0.58 N/mm^2^ and 0.62 N/mm^2^, respectively, owing to the oxidation of the wood constituents. The oxidation of wood treated with air caused more brittle features than those of the untreated wood, which was also observed in the SEM micrographs. The hardness of O1 and O2 increased from 0.50 N/mm^2^ to 0.59 N/mm^2^ and 0.57 N/mm^2^, respectively. The hardness of O2 represents high deviation and thus a lower heat treatment intensity should be applied for oil heat treatment when the wood is to be used in structural circumstances. This was most likely caused by the rearrangement of the lignin-cellulose microfibril structure and matrix system modification of wood cell walls^12,42^. Heat treatment generally caused the cellulose to tighten, rearrangement of lignin, better connections between cellulose and lignin^3^, and the formation of new lignocellulose network structures^43^.

**Figure 9:**
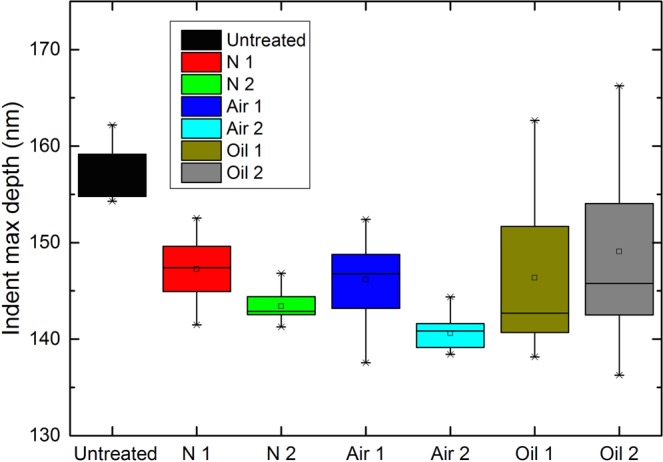
The maximum indent depth of wood samples after different heat treatment processes.

To study cell wall creep behavior from the nanoindentation measurements, load displacement and time data were obtained during the holding step of the test^44^. This approach was consistent with the constant load method reported in many studies^45–47^. Figure 9 shows the maximum indent depths of the heat-treated wood samples. After heat treatment, the same load functions were performed, and the indent depth of the wood cell wall was reduced from 157.7 nm to 140.4–149.1 nm, which proved that high-temperature heat treatment could effectively improve the cell wall hardness. The maximum indent depths of O1 and O2 showed large deviations, which was likely due to the migration of canola oil in the composite middle lamellas.

**Figure 10:**
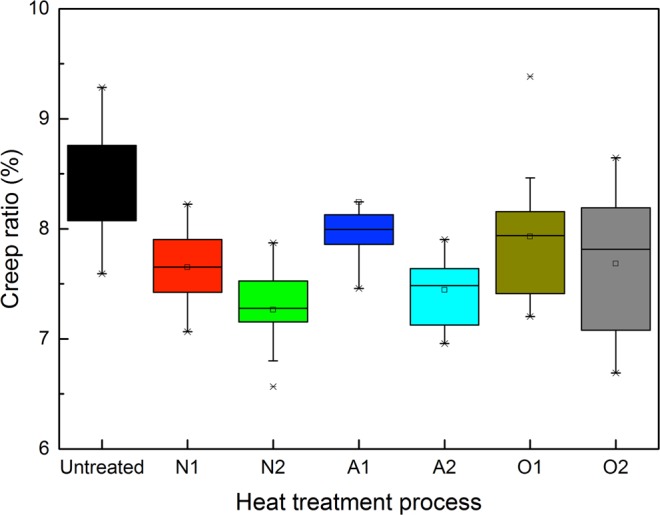
The creep ratio of different heat-treated wood samples.

As shown in Fig. 10, the creep ratio of the heat-treated wood decreased from 8.44% to 7.26–8.24%. In other words, the treatment increased the toughness of the wood cell walls. Based on the observations, heat treatment under a nitrogen atmosphere clearly reduced the creep behavior of the cell walls^48,49^, and the O1 and O2 samples represented higher deviations, which was in direct relation to the presence of oil in the cell walls. The lower creep ratio of the heat-treated wood was most likely caused by the higher reinforcement of recondensation and cross-linking reactions of the lignocellulosic structures^50,51^.

## Conclusions

Wood samples heat treated under different media were tested by nanoindentation at room temperature. The evaluation of the chemical composition, surface morphology, and micromechanical properties all demonstrated that the different heating mediums had obviously different influences on the wood properties. The results showed that degradation increased with increasing treatment temperature. Due to oil uptake, the mass of the oil heat-treated samples increased obviously, and triglyceride fatty acids were also found in the oil heat-treated samples by FTIR measurements. Heat treatment induced a series of complicated chemical reactions, such as deacetylation, depolymerization, cross-linking and recondensation, which, consequently, resulted in cracks in the cell walls. Heat treatment enhanced the hardness of the cell walls, which may be attributed to the condensation and cross-linking reactions of lignin and the modified cellulose-lignin structure. The wood cell walls represented lower creep behaviors after heat treatment, which is another notable feature of heat-treated wood.
